# Effects of RXR Agonists on Cell Proliferation/Apoptosis and ACTH Secretion/*Pomc* Expression

**DOI:** 10.1371/journal.pone.0141960

**Published:** 2015-12-29

**Authors:** Akiko Saito-Hakoda, Akira Uruno, Atsushi Yokoyama, Kyoko Shimizu, Rehana Parvin, Masataka Kudo, Takako Saito-Ito, Ikuko Sato, Naotaka Kogure, Dai Suzuki, Hiroki Shimada, Takeo Yoshikawa, Ikuma Fujiwara, Hiroyuki Kagechika, Yasumasa Iwasaki, Shigeo Kure, Sadayoshi Ito, Akira Sugawara

**Affiliations:** 1 Department of Molecular Endocrinology, Tohoku University Graduate School of Medicine, Sendai, Miyagi, Japan; 2 Department of Medical Biochemistry, Tohoku University Graduate School of Medicine, Sendai, Miyagi, Japan; 3 Division of Nephrology, Endocrinology and Vascular Medicine, Tohoku University Graduate School of Medicine, Sendai, Miyagi, Japan; 4 Department of Pediatrics, Tohoku University Graduate School of Medicine, Sendai, Miyagi, Japan; 5 Department of Pharmacology, Tohoku University Graduate School of Medicine, Sendai, Miyagi, Japan; 6 Institute of Biomaterials and Bioengineering, Tokyo Medical and Dental University, Chiyoda-ku, Tokyo, Japan; 7 Health Service Center, Kochi University, Kochi, Kochi, Japan; University of Texas at Austin Dell Medical School, UNITED STATES

## Abstract

Various retinoid X receptor (RXR) agonists have recently been developed, and some of them have shown anti-tumor effects both *in vivo* and *in vitro*. However, there has been no report showing the effects of RXR agonists on Cushing’s disease, which is caused by excessive ACTH secretion in a corticotroph tumor of the pituitary gland. Therefore, we examined the effects of synthetic RXR pan-agonists HX630 and PA024 on the proliferation, apoptosis, ACTH secretion, and pro-opiomelanocortin (*Pomc*) gene expression of murine pituitary corticotroph tumor AtT20 cells. We demonstrated that both RXR agonists induced apoptosis dose-dependently in AtT20 cells, and inhibited their proliferation at their higher doses. Microarray analysis identified a significant gene network associated with caspase 3 induced by high dose HX630. On the other hand, HX630, but not PA024, inhibited *Pomc* transcription, *Pomc* mRNA expression, and ACTH secretion dose-dependently. Furthermore, we provide new evidence that HX630 negatively regulates the *Pomc* promoter activity at the transcriptional level due to the suppression of the transcription factor *Nur77* and *Nurr1* mRNA expression and the reduction of Nur77/Nurr1 heterodimer recruiting to the *Pomc* promoter region. We also demonstrated that the HX630-mediated suppression of the *Pomc gene* expression was exerted via RXRα. Furthermore, HX630 inhibited tumor growth and decreased *Pomc* mRNA expression in corticotroph tumor cells in female nude mice *in vivo*. Thus, these results indicate that RXR agonists, especially HX630, could be a new therapeutic candidate for Cushing’s disease.

## Introduction

Cushing’s disease is caused by excessive ACTH secretion in a corticotroph tumor of the pituitary gland, or rarely corticotroph hyperplasia, and is the most frequent cause of endogenous hypercortisolism. Surgical removal of the tumor is the current first line therapy, leading to remission in 70–90% of cases. However, the risk of recurrence of Cushing’s disease reaches 20–25% at 10 years after surgery [1]. Radiation therapy is considered as a second-line option for patients with persistent and/or recurrent Cushing’s disease. However, it also has problems, including the delay of therapeutic effect or the risk of secondary hypopituitarism. Although several medical therapies have been evaluated for Cushing’s disease, including somatostatin analogues, dopamine agonists, cyproheptadine, and sodium valproate, the effects of these drugs are limited, and medical therapies are not yet considered as a standard therapy [2]. Therefore, the development of an effective and safe medical therapy is urgently required.

Retinoids are natural and synthetic vitamin A derivatives that regulate a variety of important cellular functions, including growth, differentiation, survival, and death. Retinoids exert their effects through retinoic acid receptors (RARα, β, γ) and retinoid X receptors (RXRα, β, γ), which are members of the nuclear steroid/thyroid hormone receptor superfamily [3]. Several studies have shown that a natural RAR agonist, all-*trans* retinoic acid (ATRA), or both a natural RAR and RXR agonist, 9-*cis* retinoic acid (9cRA; an isomer of ATRA), inhibited cell proliferation and ACTH secretion in ACTH-secreting tumor cells [4, 5], or reduced the tumor size in dogs with Cushing’s disease [6], suggesting that RA might exert novel therapeutic effects for Cushing’s disease [7]. Moreover, beneficial effects of RA on patients with Cushing’s disease have also been reported [8]. However, we recently observed that a synthetic RARα/β agonist, Am80, increased ACTH secretion by inducing transcription of pro-opiomelanocortin (*Pomc*) gene in murine pituitary corticotroph tumor AtT20 cells [9]. Therefore, the therapeutic effects of retinoids against Cushing’s disease remain unclear.

RXRs were initially identified as heterodimeric partners of RARs, thyroid hormone receptors (TRs), and vitamin D nuclear receptors (VDRs). The dimeric receptors with RXRs exert multiple transcriptional activities by binding to specific DNA response elements of target genes [10]. There are three types of RXR dimers: RXR homodimer, permissive heterodimers (e.g., peroxisome proliferation-activated receptor; PPAR, liver X receptor; LXR, and pregnane X receptor; PXR), and non-permissive heterodimers (e.g., RAR, VDR, and TR) [11, 12]. RXR homodimer and permissive heterodimers are activated upon RXR agonist binding. On the other hand, non-permissive heterodimers cannot be activated by the RXR agonist but only by the agonist of the partner receptor (e.g., RA, vitamin D, and thyroid hormone), although association with RXR is necessary for high-affinity DNA binding of receptors.

Various RXR selective agonists, which are also called rexinoids, have been developed, and some of them have shown anti-tumor effects both *in vivo* and *in vitro* [3, 13–16]. However, there is no report showing the effects of RXR agonists on Cushing’s disease. In the present study, we evaluated the effects of RXR on cell proliferation, apoptosis, ACTH secretion, and *Pomc* expression in AtT20 cells using synthetic RXR pan-agonists HX630 and PA024 [17–20]. We also examined the molecular mechanisms of *Pomc* gene transcription regulation by HX630 as well as the effect of HX630 on corticotroph tumor cells transplanted into female nude mice *in vivo*.

## Materials and Methods

### Reagents

RXR pan-agonists HX630 and PA024 and RARα/β agonist Am80 were previously described [9, 17–20]. Each RXR and RAR agonist was dissolved in DMSO at 10 mM and stored at -20°C. These stocks were diluted with medium to the desired concentration immediately before each experiment, keeping the final concentration of DMSO at 0.1%.

### Plasmids

Subcloned chimeric constructs containing the rat *Pomc* genomic DNA and luciferase cDNA (pGL3-Basic, Promega, Madison, WI) were used for the transient transfection studies: r*Pomc*-Luc (harboring the rat *Pomc* gene 5’-flanking region from -703 to +58 relative to the transcription start site upstream of the luciferase cDNA in pGL3-Basic), -429 to +58-luc; -379 to +58-luc; -359 to +58-luc; -293 to +58-luc; -169 to +58-luc; -12 to +58-luc [9]. β-galactosidase control plasmid in pCMV (pCMV-β-gal) was purchased from Clontech (Mountain View, CA). Murine Nurr1 and Nur77 cDNA were cloned by PCR from AtT20 cells and were subcloned into the pcDNA3 expression vector (Invitrogen, Carlsbad, CA) (Nurr1-pcDNA3 and Nur77-pcDNA3). Murine RXRα cDNA previously subcloned into pcDNA1/Amp expression vector (Invitrogen, Carlsbad, CA) (RXRα-pcDNA1/Amp) [21] was also used. In some experiments, human RARα cDNA subcloned into pCMX expression vector (pCMX-hRARα) [22] and PT109 luciferase reporter plasmids containing either direct repeat (DR)1 sequence (AGCTACTTATTG AGGTCA G AGGTCA AGTTACG) or DR5 sequence (AGCTACTTATTG AGGTCA CACTG AGGTCA AGTTACG) fused upstream of a viral thymidine kinase promoter (termed DR1-Luc and DR5-Luc, respectively) were used (the consensus half-site sequence AGGTCA is underlined) [23].

### Cell culture

Murine pituitary corticotroph tumor AtT20 cells [24] and African green monkey kidney CV-1 cells [22] were grown with Dulbecco's modified Eagle medium (DMEM) supplemented with 10% fetal bovine serum (FBS), 100 U/mL penicillin and 100 μg/mL streptomycin. Cells were cultured in a humidified incubator at 37°C with 5% CO_2_. Both cells were obtained from the American Type Culture Collection (AtT20: CCL-89, CV-1: CCL-70).

### Proliferation assay

The cell numbers were counted using a Cell Counting Kit-8 (Dojindo, Kumamoto, Japan) according to the manufacturer’s instructions. Briefly, either AtT20 cells or CV-1 cells (5×10^3^ cells/well) seeded in 96-well plates were incubated in 100 μl regular media for several days. The cells were then re-fed with DMEM supplemented with 1% resin and charcoal-treated (stripped) FBS media containing appropriate concentrations of each RXR agonist. After incubation for 96 hr, 10 μl of assay reagent were added onto each well and the plate was incubated for 4 hr at 37°C, 5% CO_2_. The generation of colored formazan product, which was bio-reduced from tetrazolium compound in metabolically active cells, was assessed optically by measuring the absorbance at 450 nm (reference 600 nm) using a microplate reader. Results are expressed as percentages of each control.

### Apoptosis assay

Cell apoptosis was estimated using a Homogeneous Caspases Assay, (fluorimetric) kit (Roche, Mannheim, Germany) according to the manufacturer’s instructions. Briefly, either AtT20 cells or CV-1 cells (5×10^3^ cells/well) seeded in 96-well plates were incubated in 100 μl regular media for several days. The cells were then re-fed with DMEM supplemented with 1% stripped FBS media containing appropriate concentrations of each RXR agonist. After incubation for 96 hr, 100 μl of substrate solution was added onto each well, and the plates were incubated for 2 hr at 37°C, 5% CO_2_. Activated caspases (2, 3, 6, 7, 8, 9, and 10) from apoptotic cells were detected by measuring the fluorescence using a microplate fluorescence reader with a 485 nm excitation filter and 535 nm emission filter. Results are expressed as percentages of each control.

### RNA isolation

AtT20 cells grown to 50% confluence in regular medium in 12-multiwell plates were incubated either without or with each RXR agonist at appropriate concentrations in DMEM supplemented with 1% stripped FBS media for 48 hr. In the overexpression experiments, each expression vector was transfected for 24 hr before treatment with HX630. The cells were then lysed and their total RNAs were isolated using NucleoSpin RNA II (TaKaRa Bio, Ohtsu, Japan) according to the manufacturer’s instructions. Samples were treated with DNase before the experiments in order to remove any contaminant DNA. The RNA was quantified by a Nanodrop 2000 (Thermo Scientific, Waltham, MA). The quality of the RNA for microarray was evaluated by an Agilent BioAnalyzer 2100. RNA samples with RIN (RNA integrity number) >8.0 and A260/A280 of approximately 2.0 were used for the gene expression analysis.

### Quantitative real-time PCR

Reverse transcription-polymerase chain reaction (PCR) was performed using TaKaRa PrimeScript RT reagent Kit with gDNA Eraser (TaKaRa Bio). Briefly, after removing genome DNA with gDNA Eraser, total RNA was converted to cDNA at 37°C for 15 min with reverse transcriptase, oligo dT primer and random 6 mers. Reverse transcription mixtures were subjected to quantitative real-time PCR with iQ Supermix (for *Pomc*) or iQ SYBR green Supermix (for others) (Bio-Rad, Hercules, CA) using a DNA Engine thermal cycler attached to a Chromo4 detector (Bio-Rad). The following primer sequences were used: mouse *Pomc* (forward, 5’-CAGTGCCAGGACCTCACC-3’, reverse, 5’-CAGCGAGAGGTCGAGTTTG-3’), mouse *RXRα* (forward, 5’-GGCTTCGGGACTGGTAGCC-3’, reverse, 5’-GCGGCTTGATATCCTCAGTG-3’), mouse *RXRβ* (forward, 5’-TGGCCACTGGCATGAAAAGG-3’, reverse, 5’-CATCTCCATCCCCGTCTTTG-3’), mouse *RXRγ* (forward, 5’-TCCTCCAGGAATCAACTTGG-3’, reverse, 5’-CTGCTGACACTGTTGACCAC-3’), mouse *NeuroD1* (forward, 5’-ACGCAGAAGGCAAGGTGTCC-3’, reverse, 5’-TTGGTCATGTTTCCACTTCC -3’), mouse *Nur77* (forward, 5’-GCACAGCTTGGGTGTTGATG-3’, reverse, 5’-CAGACGTGACAGGCAGCTG-3’) [25], mouse *Nurr1* (forward, 5’-TCAGAGCCCACGTCGATT-3’, reverse, 5’-TAGTCAGGGTTTGCCTGGAA-3’) [26] and mouse *18S rRNA* (forward, 5’- CTCAACACGGGAAACCTCAC-3’, reverse, 5’-CGCTCCACCAACTAAGAACG-3’). Sequences of taqman probes for mouse *Pomc* and mouse *18S rRNA* were 5’-AGCAACCTGCTGGCTTGCATCCG-3’ and 5’-TCTCGATTCCGTGGGTGGTGGTGC-3’, respectively. Reactions were incubated at 95°C for 3 min and then were amplified using temperature parameters of 95°C for 15 sec; 60°C for 10 sec; 72°C for 20 sec. Amplifications were carried out for 40 cycles, followed by a 3 min extension at 72°C. After amplification, a melting-curve analysis was performed from 60°C to 95°C with a heating rate of 0.5°C/10 sec and a continuous fluorescence acquisition. The signals of the samples of interest were then quantified from the standard curve, and all obtained data were normalized by mouse *18S rRNA*. To confirm the amplification specificity, the PCR products from each primer pair with SYBR green were subjected to a melting curve analysis. Results are expressed as percentages of each control.

### Measurement of ACTH concentration

AtT20 cells grown to 50% confluence in regular media in 12-multiwell plates were incubated either without or with each RXR agonist at appropriate concentrations in DMEM supplemented with 1% stripped FBS for 48 hr. The ACTH concentrations in the supernatants were thereafter measured by an ACTH (Rat, Mouse) EIA Kit (Phoenix Pharmaceuticals, Burlinngame, CA) according to the manufacturer's instructions. Data were normalized by the total protein in each well to correct for well-to-well variations. Results are expressed as percentages of each control.

### Transient transfection and luciferase assay

AtT20 cells grown to 50% confluence in regular medium in 24-multiwell plates were transiently transfected with 300 ng each r*Pomc*-Luc reporter plasmid and 150 ng pCMV-β-gal using Lipofectamine LTX and Plus reagent (Invitrogen) for 24 hr according to the manufacturer's instructions. In the overexpression experiments, 100 ng of each expression vector were also transfected. The media were changed to DMEM supplemented with 1% stripped FBS, and the cells were incubated either without or with each RXR agonist at appropriate concentrations for 24 hr. They were then washed with PBS, and the cell extracts were prepared using Glo Lysis Buffer (Promega). Luciferase activity was measured using Bright-Glo reagents (Promega), and β-galactosidase activity was simultaneously measured. Data were normalized by β-galactosidase activity. In some experiments, CV-1 cells, grown in the same conditions as in AtT20 cells, were transiently transfected either with 150 ng DR1-Luc reporter plasmid, 150 ng RXRα-pcDNA1/Amp, and 50 ng pCMV-β-gal, or with 150 ng DR5-Luc reporter plasmid, 100 ng RXRα-pcDNA1/Amp, 100 ng pCMX-hRARα, and 50 ng pCMV-β-gal. Thereafter, the DR1-Luc transfected cells were incubated with various concentrations of HX630 or PA024 for 48 hr, and the DR5-Luc transfected cells were incubated in the absence or presence of 10 μM Am80 with various concentrations of HX630 or PA024 for 48 hr.

### Chromatin immunoprecipitation (ChIP) assay with transiently transfected cells

ChIP assay was performed with the EpiQuik chromatin immunoprecipitation kit (Epigentek, Brooklyn, NY). Briefly, cells were transfected overnight with r*Pomc*-Luc reporter plasmid (-703 to +58-luc) using Lipofectamine LTX and Plus reagent (Invitrogen). Thereafter, 6×10^6^ AtT20 cells cultured for 24 hr in the presence (10 μM) or absence of HX630 were trypsinized and washed with PBS and then cross-linked in medium containing 1% formaldehyde for 10 min at room temperature. The reaction was stopped by the addition of glycine (final concentration 125 mM). The cells were washed in ice-cold PBS and lysed in a lysis buffer containing a protease inhibitor cocktail. Chromatin was sheared to an average size of 200–1,000 bp by sonication (15 pulses of 20 sec at level 2; Branson Sonifier-250). An aliquot of cross-linked DNA-containing lysates was removed as input DNA. Anti-Nurr1/Nur77 antibody (Santa Cruz Biotechnology), anti-NeuroD antibody (Cell Signaling), or normal rabbit IgG (Santa Cruz Biotechnology) was coated onto 96-well plates for 90 min at room temperature. Lysates were then added, followed by incubation for 90 min at room temperature on a rocking platform. The cross-linked DNA fragments bound to the plates were washed and reversed by proteinase K treatment. Precipitated DNA fragments were recovered by column purification, and conventional PCR reactions were performed. The following specific primers were designed to amplify a 211 bp sequence (-447 to -237 region) including the Nur-responsive element (NurRE; -404/-383) and E-box (-377/-370) (forward: 5′-ACACTGGGGAAATCTGATGC-3′, reverse: 5′-CGGTGGTCAGGAGGAACTTA-3′) in the rat *Pomc* promoter region. Thermal cycles included 3 min at 95°C, followed by 35 cycles of 95°C for 1 min, 60°C for 1 min, and 72°C for 1 min. PCR products were analyzed by agarose gel electrophoresis.

### Small interfering RNA

Small interfering RNAs (siRNAs) for RXRα (SI01409051) and negative control siRNA (1027280) were obtained from Qiagen (Hilden, Germany). AtT20 cells grown to 50% confluence in 24-multiwell plates were transiently transfected with 10 pmol siRNAs using Lipofectamine 2000 reagent (Invitrogen) for 48 hr according to the manufacturer's instructions. For the luciferase assay, reporter plasmids were also transfected. The cells were then incubated either without or with 10 μM HX630 for 24 hr. Thereafter they were used for luciferase assay or quantitative RT-PCR.

### Microarray analyses

Microarray analyses were performed using the Whole Mouse Genome Oligo Microarray (Mouse GE 4x44K v2 Microarray Kit; G4846A) (Agilent Technologies, Santa Clara, CA) [27]. A full list of cDNAs is available online (www.agilent.com). Protocols for sample preparation and hybridization of the mononuclear cells were adaptations of those in the Agilent Technical Manual. Briefly, Cyanine-3 (Cy3) labeled cRNA was prepared from 200 ng RNA using a Low Input Quick-AMP labeling kit (Agilent Technologies) according to the manufacturer's instructions, followed by RNAeasy column purification (QIAGEN). Dye incorporation and cRNA yield were checked with a NanoDrop spectrophotometer. Thereafter, Cy3-labelled cRNA was fragmented at 60°C for 30 min. Fragmented cRNA samples were hybridized onto chips by means of 17 hr of incubation at 65°C with constant rotation, followed by a two-step microarray wash of 1 min in two washing buffers (Agilent Technologies). Hybridized microarrays were scanned in an Agilent Technologies Scanner (model G2505C), and the scanned images were analyzed with Feature Extraction Software 10.7.3.1 (Agilent) using default parameters (protocol GE1_107_Sep09 and Grid: 026655_D_F_20100123) to obtain background subtracted and spatially de-trended Processed Signal intensities.

### Microarray data analyses

The microarray data were analyzed using GeneSpring software version 12.0 (Agilent Technologies) [27], and the analysis was conducted by comparing between 10 μM HX630 and the control in AtT20 cells. A fold-change of >1.5 or <-1.5 and *P*-value<0.01 in a T-test statistical analysis were used as the criteria for significant gene expression changes. This resulted in the identification of 5,342 genes with significant changes in expression between 10 μM HX630 and control. A data set containing gene identifiers (5,342 differentially expressed genes) and corresponding expression values was then uploaded into the Ingenuity Pathway Analysis (IPA) software (Ingenuity Systems, Redwood City, CA; www.ingenity.com) for functional and pathway analyses. Each gene identifier was mapped to its corresponding object in the Ingenuity^®^ Knowledge Base. These genes, called Network Eligible molecules, were overlaid onto a global molecular network developed from information contained in the Ingenuity Knowledge Base. Networks of Network Eligible Molecules were then algorithmically generated based on their connectivity. The Functional Analysis identified the biological functions and/or diseases that were most significant to the data set. Genes from the data set that were associated with biological functions and/or diseases in the Ingenuity Knowledge Base were considered for the analysis. The Functional Analysis of a network identified the biological functions and/or diseases that were most significant to the molecules in the network. The network molecules associated with biological functions and/or diseases in the Ingenuity Knowledge Base were considered for the analysis. Fisher’s exact test was used to calculate the *P*-value (<0.05) determining the probability that each biological function and/or disease assigned to that data set or network could be due to chance alone. The Z-score was calculated by weighted Z-tests and >2 or <2 showed significant difference. Canonical pathways analysis identified the pathways from the IPA library of canonical pathways that were most significant to the data set. Molecules from the data set that were associated with a canonical pathway in the Ingenuity Knowledge Base were considered for the analysis. The significance of the association between the data set and the canonical pathway was measured in 2 ways: 1) A ratio of the number of molecules from the data set that map to the pathway divided by the total number of molecules that map to the canonical pathway is displayed. 2) Fisher’s exact test was used to calculate the *P*-value (<0.05) (–Log (*P*-value) >1.3) determining the probability that the association between the genes in the dataset and the canonical pathway can be explained by chance alone. All microarray data in this study have been deposited in NCBI's Gene Expression Omnibus (GEO) and are accessible through GEO Series accession number GSE43783 (http://www.ncbi.nlm.nih.gov/geo/query/acc.cgi?acc=GSE43783).

### *In vivo* tumor formation

Eight-week-old female BALB/c-nu mice (nu/nu) were subcutaneously inoculated with AtT20 cells (1,000,000/mouse). The mice were housed in temperature- and humidity-controlled cages with a 12-hour/12-hour light/dark cycle, and were given standard chow diet and tap water *ad libitum*. After 1 week, the animals were randomized either for the HX630 (5 mg/kg/day) injection or for the vehicle (corn oil) injection intraperitonealy 3 times a week for 3 weeks. Their body weights and the tumor volumes were measured throughout the treatment. The tumor volume was measured as previously described [5]. Briefly, a vernier was used to measure the short and long axis of the tumor and the following formula was used: tumoral volume = [(short axis) ^2^ × long axis × 0.4]. Thereafter, the mice were euthanized and their tumors were removed to isolate total RNAs for *Pomc* mRNA expression analyses. Blood samples were also collected from the mice for the measurement of their plasma ACTH levels. All animal experiments were performed in accordance with the institutional regulations for animal care of Tohoku University Graduate School of Medicine. The protocol was approved by the Committee on the Ethics of Animal Experiments of the Tohoku University. Animals were sacrificed by sevoflurane anesthesia, and all efforts were made to minimize suffering.

### Statistical analysis

All data except for microarray analysis are presented as mean ± SEM. Statistical analyses were performed with ANOVA followed by post hoc Tukey test. *P*<0.05 was considered statistically significant.

## Results

### Effects of RXR agonists on cell proliferation and apoptosis in AtT20 cells

**Fig 1:**
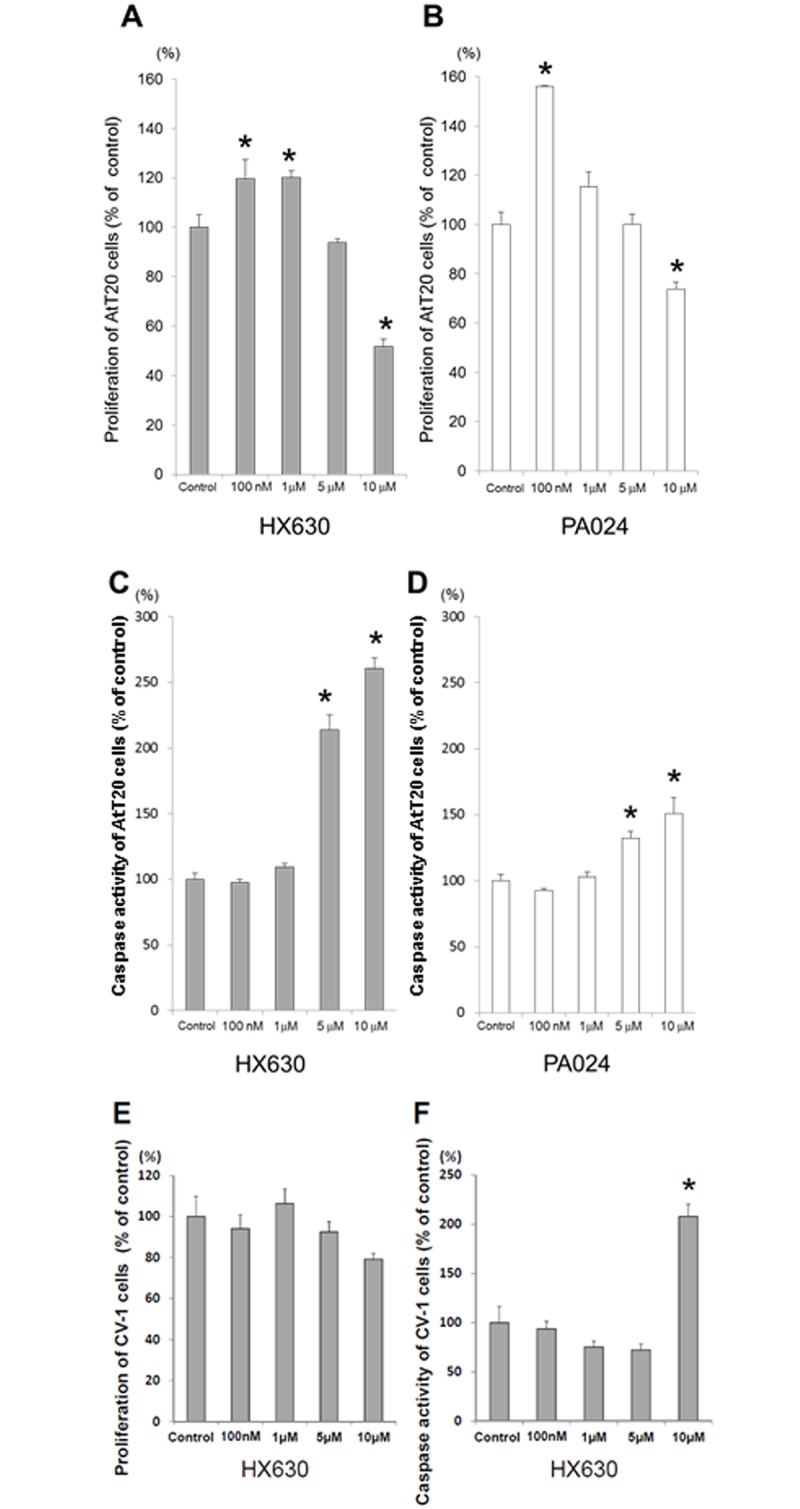
Effects of RXR agonists on AtT20 cell/CV-1 cell proliferation and apoptosis. AtT20 cells were incubated for 96 hr either in the presence (100 nM, 1 μM, 5 μM, or 10 μM) or absence (control) of each RXR agonist, HX630 and PA024, respectively, before each assay. Effects of each RXR agonist on proliferation and apoptosis are shown in A, B and C, D, respectively. CV-1 cells were also incubated for 96 hr either in the presence or absence of HX630 as in AtT20 cells, and their proliferation (E) and apoptosis (F) were thereafter assayed. Results are expressed as percentages of each control. Each point represents mean ± SEM (n = 6). *P<0.05 vs control of each RXR agonist.

We examined the effects of RXR agonists on AtT20 cell proliferation using a WST-8 assay after incubation with various concentrations of agonists for 96 hr. RXR pan-agonists HX630 and PA024 significantly suppressed AtT20 cell proliferation at 10 μM (Fig 1A and 1B). We next examined the effects of RXR agonists on the apoptosis of AtT20 cells by activated caspase assay. HX630 and PA024 induced the apoptosis of AtT20 cells dose-dependently (Fig 1C and 1D). These data indicated that the inhibitory effects of HX630 and PA024 on AtT20 cell proliferation were caused by inducing apoptosis. In order to examine the effect of HX630 on other cell lines, we treated CV-1 cells with various concentrations of HX630. As shown in Fig 1E, HX630 did not affect the cell proliferation of CV-1 cells. Additionally, HX630-induced CV-1 cell apoptosis was observed only at 10 μM (Fig 1F). Taken together, the strong effects of HX630 on the inhibition of cell proliferation and the induction of apoptosis may be especially prominent in AtT20 cells.

### *RXRα*, *β*, and *γ* mRNA expression in AtT20 cells

**Fig 2:**
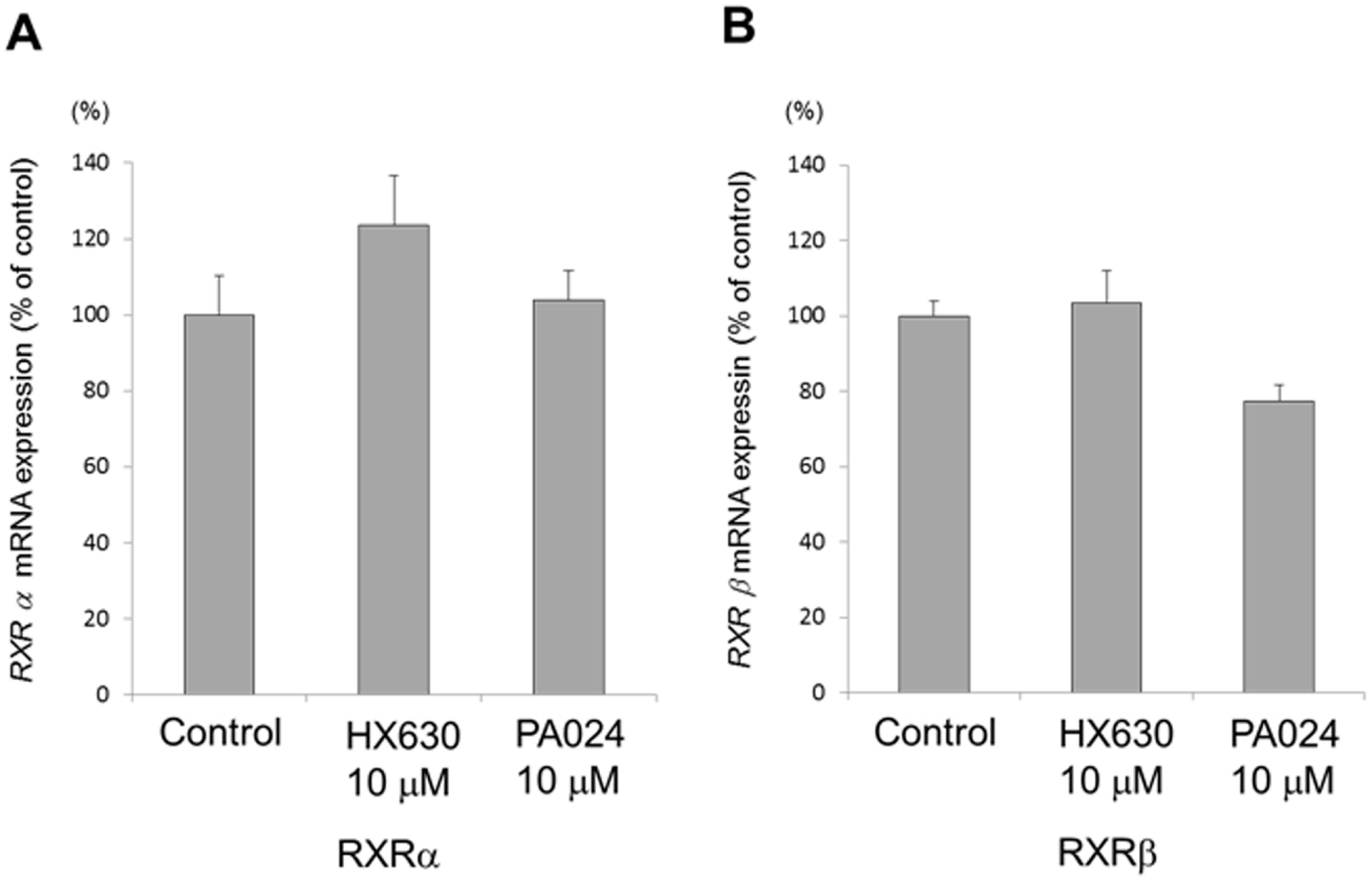
*RXRα* and *β* mRNA expression in AtT20 cells, and the effects of HX630 and PA024 on them. In A and B, total RNAs extracted from the culture for 48 hr either in the presence (10 μM) or absence (control) of each RXR agonist, HX630 and PA024, respectively, were subjected to examine *RXRα* and *β* mRNA expression by quantitative real-time PCR. Results are expressed as percentages of each control. Each point represents mean ± SEM (n = 4).

The expression of *RXR* isoform mRNA in AtT20 cells was confirmed by quantitative real-time PCR. *RXRα* and *β* mRNAs were expressed in AtT20 cells (Fig 2A and 2B), while *RXRγ* mRNA was not detected consistent to our previous observation [28]. Neither HX630 nor PA024 at 10 μM affected the *RXRα* or *β* mRNA expression levels.

### Effects of RXR agonists on *Pomc* mRNA expression, ACTH secretion, and *Pomc* promoter activity in AtT20 cells

**Fig 3:**
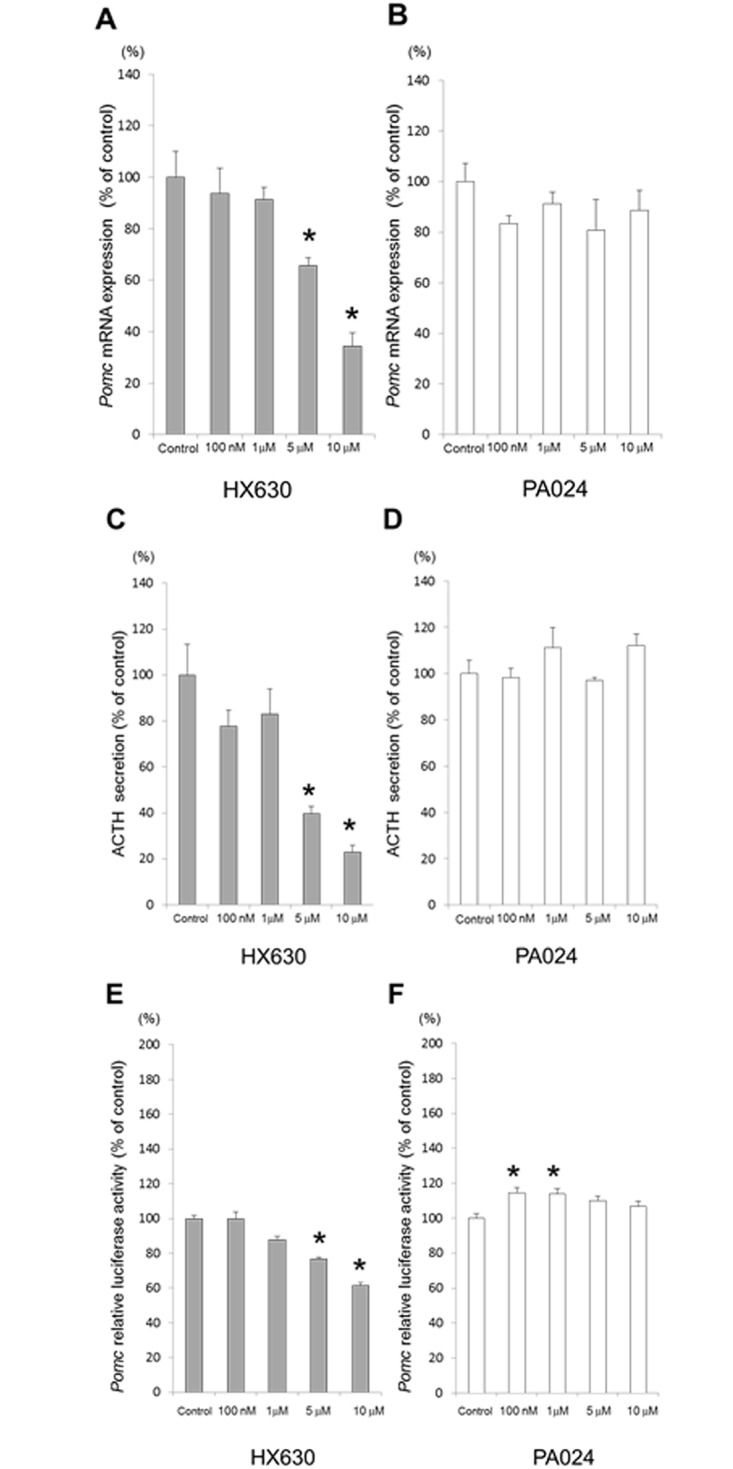
Effects of RXR agonists on *Pomc* mRNA expression, ACTH secretion, and *Pomc* promoter activity in AtT20 cells. In A and B, total RNAs extracted from the cells cultured for 48 hr either in the presence (100 nM, 1 μM, 5 μM, or 10 μM) or absence (control) of each RXR agonist, HX630 and PA024, respectively, were examined for *Pomc* mRNA expression by quantitative real-time PCR. In C and D, supernatants obtained from the cells cultured for 48 hr either in the presence (100 nM, 1 μM, 5 μM, or 10 μM) or absence (control) of each RXR agonist, HX630 and PA024, respectively, were examined for ACTH secretion by EIA. In E and F, AtT20 cells transiently transfected for 24 hr with r*Pomc*-luc (-703 to +58-luc) and pCMV-β-gal were incubated either in the presence (100 nM, 1 μM, 5 μM, or 10 μM) or absence (control) of each RXR agonist, HX630 and PA024, respectively, before the luciferase assay. Results are expressed as percentages of each control. Each point represents mean ± SEM (n = 4). *P<0.05 vs control of each RXR agonist.

We examined the effects of RXR agonists on *Pomc* mRNA expression level in AtT20 cells. HX630 decreased *Pomc* mRNA expression in a dose-dependent manner, but PA024 did not affect its expression (Fig 3A and 3B). We next examined the effects of RXR agonists on ACTH secretion from AtT20 cells to their supernatants. HX630 dose-dependently decreased ACTH secretion from AtT20 cells (Fig 3C). However, PA024 did not affect its secretion (Fig 3D). Furthermore, we examined the effects of RXR agonists on the *Pomc* promoter activity. HX630 decreased the *Pomc* promoter activity in a dose-dependent manner (Fig 3E), while the activity was not affected by PA024 (Fig 3F). These data suggest that HX630 negatively regulates *Pomc* transcription, resulting in the suppression of *Pomc* mRNA expression and ACTH secretion in AtT20 cells.

### Effects of RXR agonists on the *Pomc* promoter deletion mutants

**Fig 4:**
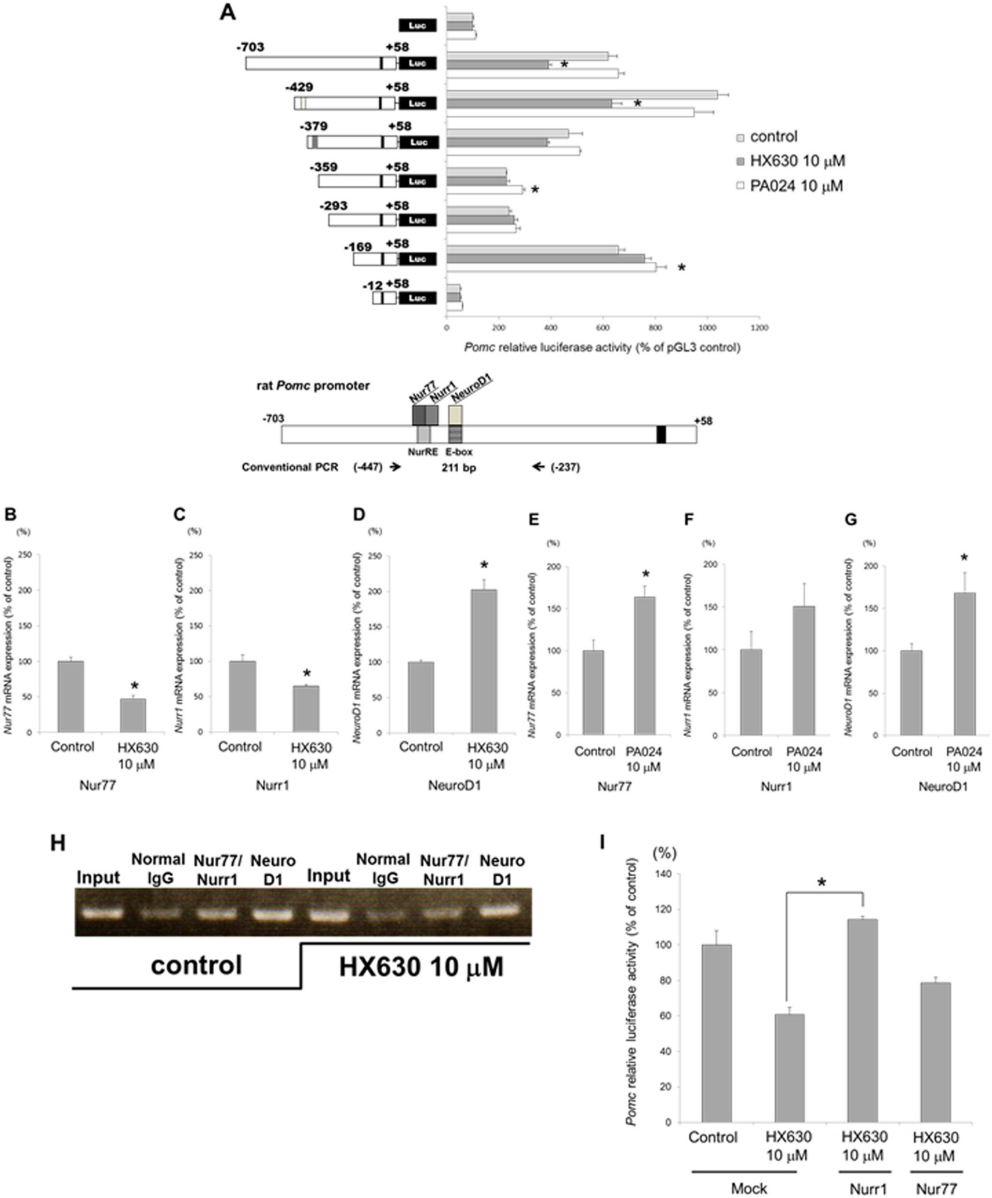
Roles of *Nur77*, *Nurr1*, and *NeuroD1* on the HX630-mediated suppression of *Pomc* promoter activity. A, effects of RXR agonists on the *Pomc* promoter deletion mutants in AtT20 cells. AtT20 cells transiently transfected for 24 hr with r*Pomc*-luc (-703 to +58-luc) or each deletion mutant reporter plasmid (-429 to +58-luc; -379 to +58-luc; -359 to +58-luc; -293 to +58-luc; -169 to +58-luc; -12 to +58-luc) and pCMV-β-gal were incubated either in the presence (10 μM) or absence (control) of each RXR agonist, HX630 and PA024, respectively, before the luciferase assay. Results are expressed as percentages of each control. Each point represents mean ± SEM (n = 4). * *P*<0.05 vs control in pGL3-Basic. B-G, effects of HX630 or PA024 on *Nur77*, *Nurr1*, and *NeuroD1* mRNA expression in AtT20 cells. Total RNAs extracted from the cells cultured for 48 hr either in the presence (10 μM) or absence (control) of HX630 (B-D) or PA024 (E-G) were subjected to examine *Nur77*, *Nurr1*, and *NeuroD1* mRNA expression by quantitative real-time PCR. Results are expressed as percentages of each control. Each point represents mean ± SEM (n = 4). *P<0.05 vs control of each RXR agonist. H, effects of HX630 on the binding of Nurr1/Nur77 heterodimer or NeuroD1 to the *Pomc* promoter. ChIP assays were performed using digested chromatin extracted from the cells cultured for 24 hr either in the absence (control) or presence (10 μM) of HX630 after transfection overnight with r*Pomc*-Luc reporter plasmid (-703 to +58-luc). Chromatin fragments were immunoprecipitated either by normal rabbit IgG (negative control), Nurr1/Nur77 antibody, or NeuroD1 antibody. Purified DNA was analyzed by conventional PCR using primers specific for both NurRE and E-box contained sequence in the rat *Pomc* promoter. The expected size for a fragment including NurRE and E-box is 211 bp (indicated in the lowest chart of Fig 4A). Few PCR products observed in the input samples were detected in the immunoprecipitation using normal rabbit IgG. I, effect of overexpression of Nurr1 or Nur77 on the HX630-mediated suppression of *Pomc* promoter activity. AtT20 cells transiently co-transfected for 24 hr with r*Pomc*-luc (-703 to +58-luc), pCMV-β-gal, Nurr1-pcDNA3, Nur77-pcDNA3, or pcDNA3 (Mock) were incubated either in the presence (10 μM) or absence (control) of HX630 for 24 hr, respectively, before luciferase assay. Results are expressed as percentages of the control. Each point represents mean ± SEM (n = 4). *P<0.05 vs Mock at 10 μM HX630.

To explore the molecular mechanisms of the *Pomc* transcription regulation, we examined the promoter activity of the *Pomc* 5’-flanking region deletion mutants. The HX630-mediated transcription suppression of the *Pomc* promoter activity observed in the 5’-flanking region from -703 to +58 relative to the transcription start site gradually diminished in parallel with the gradual deletions from -703 to -359, while PA024 did not affect the *Pomc* promoter activity of these mutants (Fig 4A). There exists two transcription factor binding elements in the *Pomc* promoter between -703 and -359 bp; one is for Nur77/Nurr1 (NurRE; -404/-383) [29, 30] and the other is for NeuroD1 (Ebox; -377/-370) [31, 32] (Fig 4A). These data therefore suggest that these transcription factors may be involved in the HX630-mediated suppression of the *Pomc* promoter activity.

### Roles of Nur77/Nurr1/NeuroD1 on the HX630-mediated suppression of *Pomc* promoter activity

We examined the effects of HX630 and PA024 on the *Nur77*, *Nurr1*, and *NeuroD1* mRNA expression levels in AtT20 cells. HX630 at 10 μM significantly decreased *Nur77* and *Nurr1* mRNA expression, while it increased *NeuroD1* mRNA expression compared with the control (Fig 4B, 4C and 4D). On the other hand, PA024 at 10μM significantly increased the *Nur77*, *Nurr1*, and *NeuroD1* mRNA expression (Fig 4E, 4F and 4G). We next employed a chromatin immunoprecipitation (ChIP) assay using transiently transfected cells with r*Pomc*-Luc reporter plasmid (-703 to +58-luc) to confirm whether HX630 could affect the recruiting of Nur77/Nurr1 heterodimer or NeuroD1 to the *Pomc* promoter. The conventional ChIP assay demonstrated that Nur77/Nurr1 heterodimer and NeuroD1 under the basal condition (control) were obviously recruited to NurRE and E-box, respectively (Fig 4H). However, 10 μM HX630 decreased the binding of Nur77/Nurr1 heterodimer to NurRE compared with the control, although it did not diminish the binding of NeuroD1 to E-box (Fig 4H). Little PCR product was observed with immunoprecipitation using normal rabbit IgG both in the absence (control) or presence of 10 μM HX630 (Fig 4H). The overexpression of Nurr1 completely, and that of Nur77 partially, abolished the HX630-mediated suppression of *Pomc* promoter activity (Fig 4I). These results suggest that the HX630-mediated suppression of *Pomc* promoter activity may possibly be due to the reduction of Nur77/Nurr1 heterodimer binding to NurRE in the *Pomc* promoter.

### Involvement of RXRα in the effect of HX630 on *Pomc* promoter activity and mRNA expression

**Fig 5:**
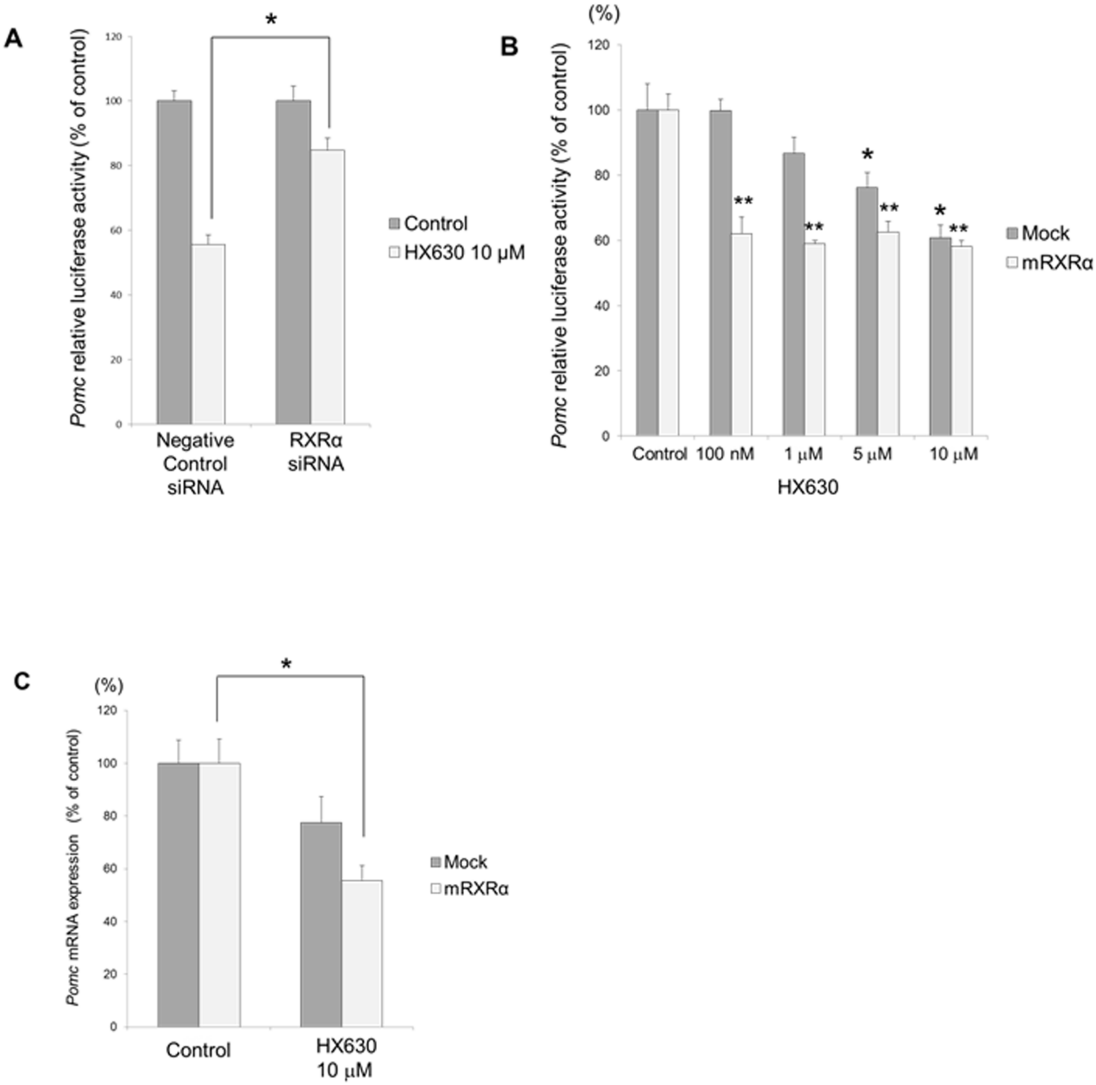
Involvement of RXRα in the HX630 effects on *Pomc* promoter activity and mRNA expression. A, effects of RXRα knockdown by its siRNA on the *Pomc* promoter activity. AtT20 cells transiently transfected with r*Pomc*-luc, pCMV-β-gal, and siRNA (negavive control or RXRα) for 48 hr were incubated either in the presence (10 μM) or absence (control) of HX630 for 24 hr, respectively. Results are expressed as percentages of each control. Each point represents mean ± SEM (n = 4). *P<0.05 vs negative control siRNA at 10 μM HX630. B, effect of RXRα overexpression on the *Pomc* promoter activity. AtT20 cells transiently transfected with r*Pomc*-Luc, pCMV-β-gal, and RXRα-pcDNA1/Amp (mRXRα) or pcDNA3 (Mock) for 24hr were incubated either in the presence (100 nM, 1 μM, 5 μM, or 10 μM) or absence (control) of HX630 for 24 hr respectively. Results are expressed as percentages of each control. Each point represents mean ± SEM (n = 4). *P<0.05 vs Mock control. **P<0.05 vs RXRα control. C, effect of RXRα overexpression on *Pomc* mRNA expression. AtT20 cells transiently transfected with RXRα-pcDNA1/Amp (mRXRα) or pcDNA3 (Mock) for 24 hr were incubated either in the presence (10 μM) or absence (control) of HX630 for 24 hr, respectively. Results are expressed as percentages of each control. Each point represents mean ± SEM (n = 4). *P<0.05 vs RXRα control.

We examined the involvement of RXRα on the *Pomc* promoter activity by knockdown using its small interfering RNA (siRNA). siRNA for RXRα significantly abolished the HX630-mediated suppression of *Pomc* promoter activity (Fig 5A). The decrease of endogenous RXRα mRNA expression by its siRNA was confirmed by quantitative real-time PCR (data not shown). On the other hand, RXRα overexpression augmented the HX630-mediated suppression of *Pomc* promoter activity, and the suppression could be observed even at a low dose (100 nM) HX630 (Fig 5B). Furthermore, RXRα overexpression augmented the HX630-mediated suppression of *Pomc* mRNA expression (Fig 5C). These data suggest that the negative regulation of *Pomc* transcription by HX630 is most likely mediated via RXRα.

### Microarray analyses of HX630-regulated gene expression

**Fig 6:**
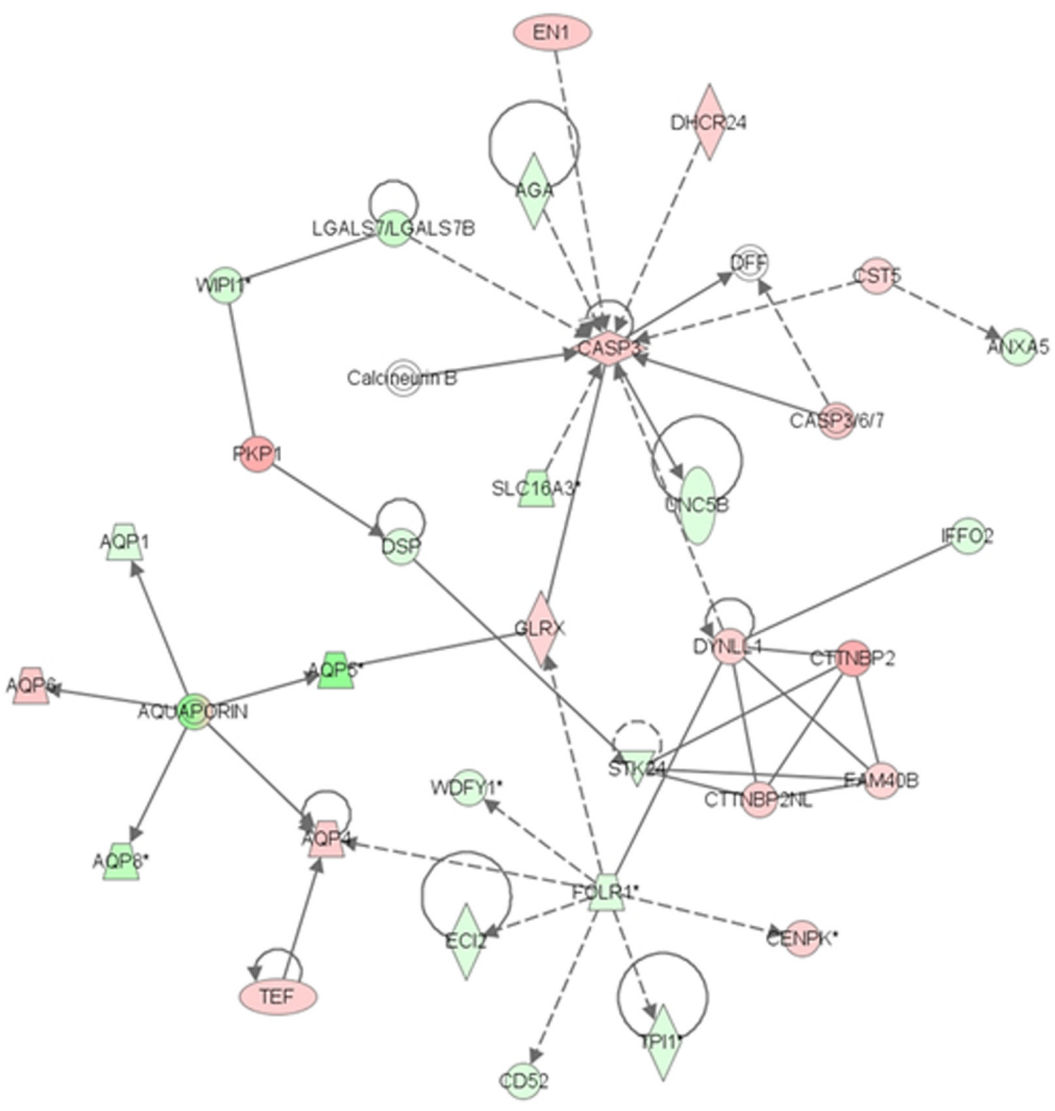
The network identified around *Casp3* in AtT20 cells treated with HX630. Molecules are represented as nodes, and the biological relationship between two nodes is represented as an edge (line). The intensity of the node color indicates the degree of up—(red) or down—(green) regulation.

To gain further insights regarding the functional mechanisms of HX630 on AtT20 cells, especially concerning cell proliferation and apoptosis, we conducted DNA microarray analyses to identify gene expression patterns, networks, and pathways in AtT20 cells treated with 10 μM HX630. A total of 2,173 up-regulated genes and 3,169 down-regulated genes, which demonstrated fold-changes of at least 1.5 compared with the control and a *P*-value<0.01 in AtT20 cells treated with 10 μM HX630, were identified. Both the differentially expressed up-regulated and down-regulated genes were subjected to IPA [27]. IPA groups significant genes according to biological processes in which they function, and identified various functions, networks, and pathways that were regulated by 10 μM HX630. Table 1 shows the categories of the biological functions and diseases significantly regulated by 10 μM HX630 that are associated with cell death, growth, and cancer. These data indicated that HX630 induced cell death and suppressed cell growth in the AtT20 cells, consistent with the results of the *in vitro* studies. Specifically, *CASP3* (caspase 3) and *CASP8* (caspase 8), which play a crucial role in apoptosis, were up-regulated, and *TNFSF10* (tumor necrosis factor ligand superfamily, member 10), which triggers the activation of caspase 3 and caspase 8, was also up-regulated. Anti-apoptotic *Bcl2* (B-cell leukemia/lymphoma 2) was down-regulated. Among tumor suppressor genes, *CDKN2B* (cyclin-dependent kinase inhibitor 2B; p15), *CDKN2C* (cyclin-dependent kinase inhibitor 2C; p18), and *BRCA2* (breast cancer 2) were up-regulated, while *CDKN1A* (cyclin-dependent kinase inhibitor 1A; p21) was down-regulated. Additionally, HX630 negatively regulated other growth regulatory biomarker genes, such as *EMP3* (epithelial membrane protein 3), *FOS* (FBJ osteosarcoma oncogene), *MAP3K8* (mitogen-activated protein kinase kinase kinase 8), *MAPK12* (mitogen-activated protein kinase 12), *PDGFC* (platelet-derived growth factor C), *PDGFRB* (platelet-derived growth factor receptor, beta polypeptide) and *TGFB3* (transforming growth factor, beta 3). On the other hand, 25 networks of significantly expressed genes regulated by 10 μM HX630 were identified (e.g. cellular movement, genetic disorder, neurological disease, and lipid metabolism). Among them, a significant gene network associated with apoptosis is presented in Fig 6. The up-regulation of caspase 3 is related to the induction of apoptosis, and these data also support the effect of HX630 on tumor suppression.

**Table 1:** The biological functions in the categories of “cell death”, “cellular growth and proliferation”, and “cancer” regulated by HX630 treatment in AtT20 cells.

Category	Functions Annotation	P-Value	Predicted Activation State	Regulation z-score	Molecules
**Cell Death**		**2.59E-10–3.23E-03**			**767**
	cell death	6.10E-10	Increased	2.018	764
	cell death of tumor cell lines	1.86E-04	Increased	2.472	288
	apoptosis of tumor cell lines	2.26E-04	Increased	2.317	247
**Cellular Growth /Proliferation**		**3.54E-07–2.59E-03**			**742**
	proliferation of cells	3.54E-07	Decreased	-3.374	603
**Cancer**		**2.53E-10–2.73E-03**			**946**
	benign tumor	5.67E-05	Decreased	-2.039	172

### Effects of HX630 on corticotroph tumor cells *in vivo*

**Fig 7:**
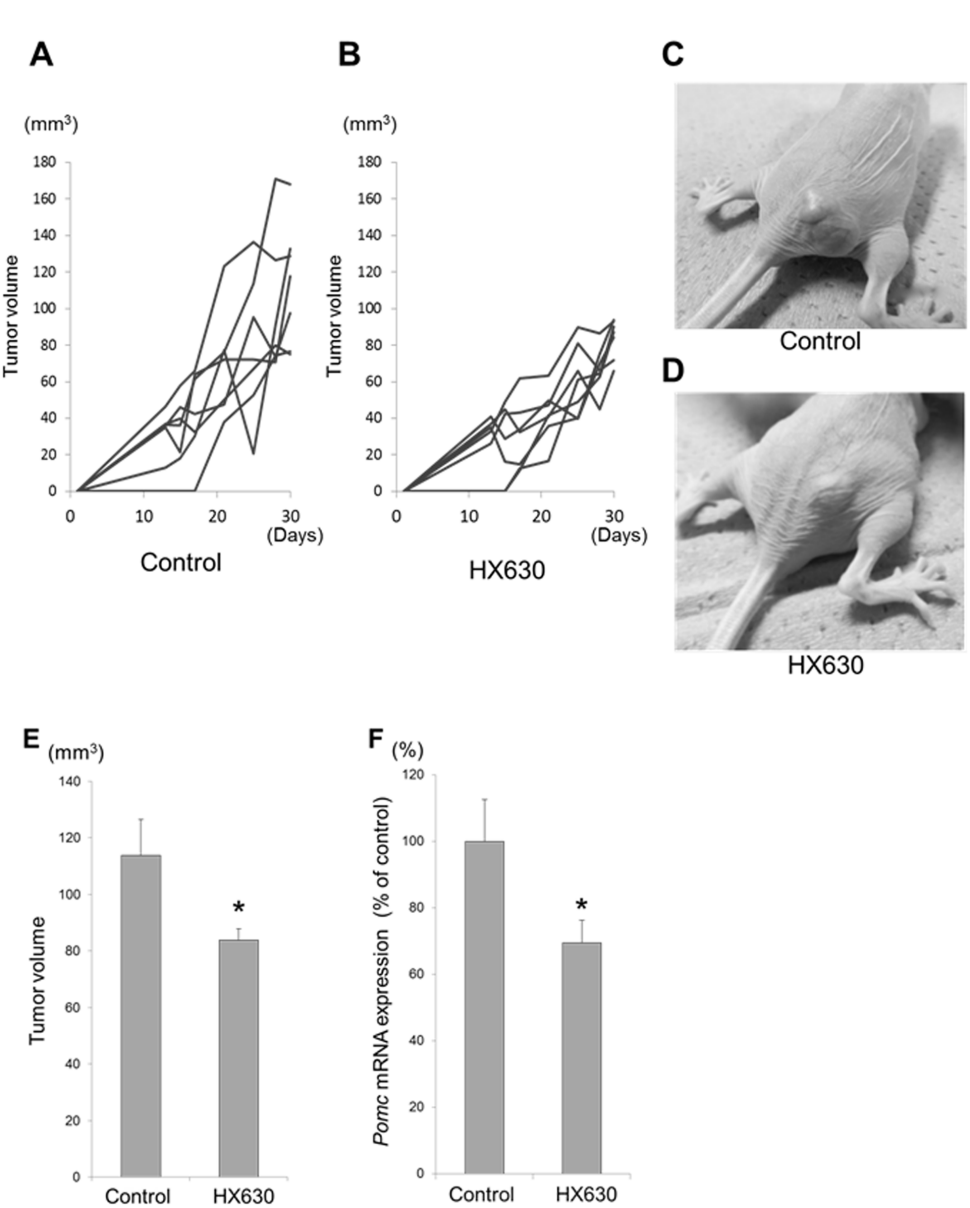
Effects of HX630 on corticotroph tumor cells *in vivo*. The mice (nu/nu) were subcutaneously inoculated with AtT20 cells (1,000,000/mouse). After 1 week, they were randomized for injection with either HX630 (5 mg/kg/d) or vehicle (corn oil) intraperitonealy 3 times a week for 3 weeks. In A and B, the body weights and the tumor volumes were monitored throughout the treatment. In C and D, a representative mouse of either the control group or the HX630-treated group on the last day is shown, respectively. Thereafter, mice were euthanized and their tumors were removed. Total RNAs were isolated from tumors and were subjected to examine *Pomc* mRNA expression by real-time PCR. In E and F, tumor volumes and *Pomc* mRNA expression in tumor cells on the last day were compared between the control group and the HX630 treated group, respectively. Results of *Pomc* mRNA expression are expressed as percentages of the control. Each point represents mean ± SEM (n = 7). *P<0.05 vs control.

To examine the anti-tumor effects of HX630 *in vivo*, we injected nu/nu mice with HX630 or vehicle intraperitonealy 3 times a week for 3 weeks after subcutaneous inoculation with AtT20 cells. Although there were no significant differences in body weights and plasma ACTH levels between the control group and the HX630 treated group (data not shown), HX630 significantly decreased the tumor volumes (Fig 7A–7E) as well as *Pomc* mRNA expressions in the tumor cells (Fig 7F). Similar observation was obtained regarding tumor volume decrease when we extended the duration of tumor cell inoculation and HX630/vehicle injection totally to 5 weeks (data not shown).

### Differential effects of HX630 and PA024 on DR1/DR5 sequence transcription

**Fig 8:**
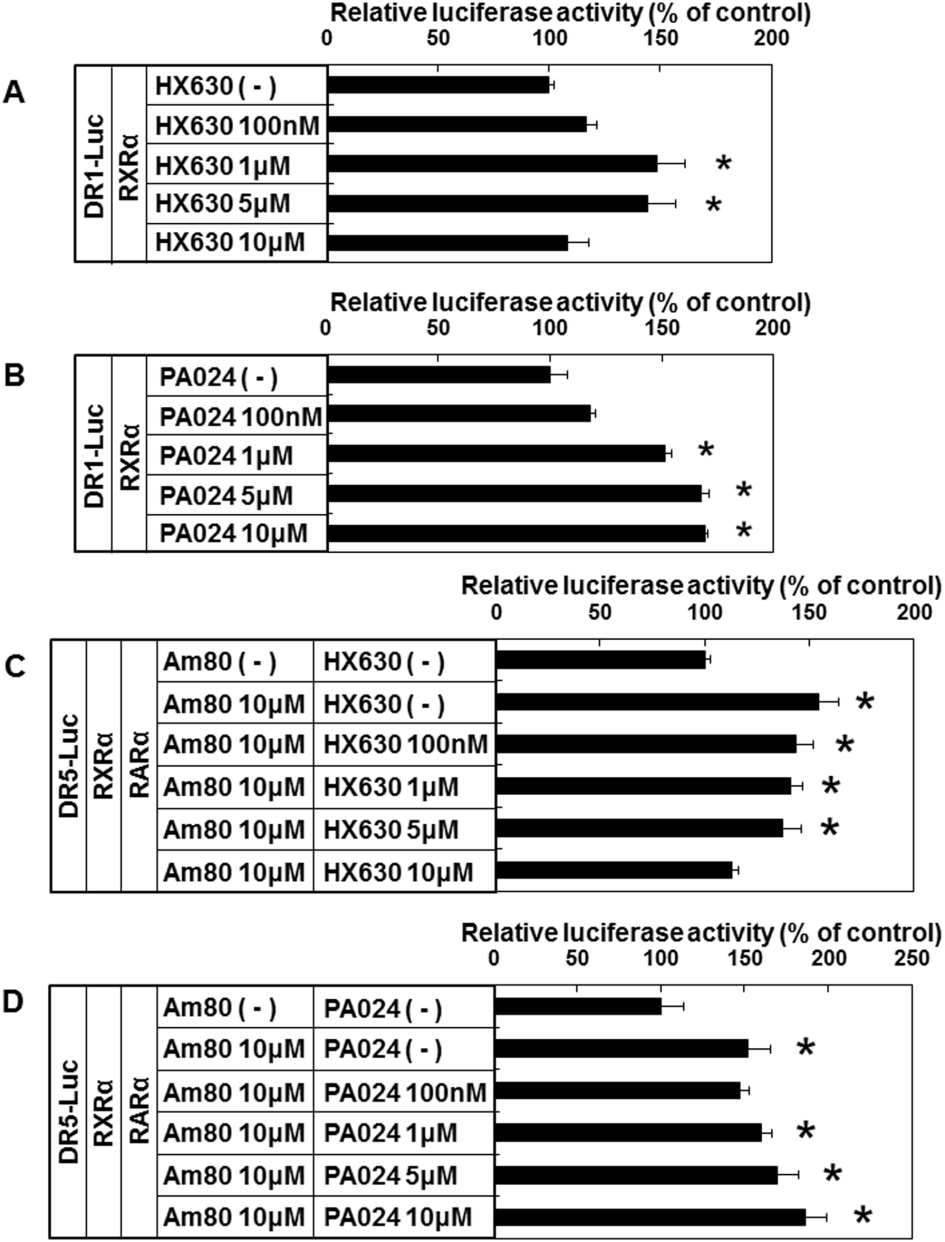
Effects of RXR agonists on DR1/DR5 sequence transcription. CV-1 cells transiently transfected with DR1-Luc reporter plasmid (DR1-Luc), RXRα-pcDNA1/Amp (RXRα), and pCMV-β-gal were incubated either without (-) or with (100 nM, 1 μM, 5 μM, or 10 μM) HX630 (A) or PA024 (B) for 48 hr. Additionally, CV-1 cells transiently transfected with DR5-Luc reporter plasmid (DR5-Luc), RXRα-pcDNA1/Amp (RXRα), pCMX-hRARα (RARα), and pCMV-β-gal were incubated either in the absence (-) or presence of 10 μM Am80 without (-) or with (100 nM, 1 μM, 5 μM, or 10 μM) HX630 (C) or PA024 (D) for 48 hr. Results are expressed as percentages of each control. Each point represents mean ± SEM (n = 4). *P<0.05 vs control (HX630 (-) in A, PA024 (-) in B, Am80 (-) HX630 (-) in C, and Am80 (-) PA024 (-) in D).

We next examined the effects of HX630 and PA024 on RXR homodimer/heterodimer using CV-1 cells. RXR homodimer activated by RXR agonists is known to stimulate DR1 sequence transcription [10, 11, 33]. On the other hand, RXR agonists can stimulate DR5 sequence transcription via RXR-RAR heterodimer only in the presence of RAR agonists (non-permissive heterodimer) [10, 11, 34]. In the presence of RXRα, HX630 stimulated DR1 sequence transcription maximally at 1 μM (Fig 8A), while PA024 stimulated DR1 sequence transcription maximally at 10 μM (Fig 8B). On the other hand, in the presence of both RARα and RXRα, Am80-stimulated DR5 sequence transcription was dose-dependently inhibited by HX630 (Fig 8C), while it was further augmented by high-dose PA024 (Fig 8D). These data indicate that HX630 and PA024 differentially affect RXR homodimer bound to DR1 sequence as well as RXR-RAR heterodimer bound to DR5 sequence in the presence of Am80.

## Discussion

RXR agonists have been expected to have therapeutic potential for cancer prevention and treatment because they are less toxic than RAR agonists [13, 14], and RXR expression is rarely lost in human tumors [12]. Indeed several RXR agonists (e.g. LG100268, AGN194204, 9-cis UAB30) are found to be effective in suppressing tumor development in multiple carcinogenesis models, including those of breast, skin, pancreas, and prostate [3, 13–16]. Furthermore, an RXR agonist, bexarotene (LGD1069), which is a novel oral synthetic derivative of 9cRA, is already approved for the treatment of refractory cutaneous T-cell lymphoma and non-small cell lung cancer in human [16, 35, 36]. The molecular mechanisms proposed for the anti-tumor effects of RXR agonists include inhibition of cell cycle progression and induction of apoptosis. RXR agonists can induce tumor cell cycle arrest by down-regulation of the cyclin D1/D3 expressions [16, 37], activation of p53 [16], and up-regulation of p21 [38]. On the other hand, RXR agonists can induce apoptosis of tumor cell lines in association with the activation of caspase-3/8, cleavage of poly-(ADP-ribose) polymerase, and down-regulation of survivin, which is a novel member of the inhibitor of apoptosis protein family [16, 39].

In the present study, we demonstrated that HX630 and PA024, both of which are RXR-selective pan-agonists, exerted anti-proliferative and pro-apoptotic effects in murine pituitary corticotroph tumor AtT20 cells at their high concentrations. These effects may be especially prominent in AtT20 cells, since HX630 less affected cell proliferation/apoptosis in CV-1 cells. Furthermore, we confirmed that HX630 inhibited tumor growth *in vivo*. RXR agonists are known to exert their effects through RXR homodimer or permissive heterodimers, not through non-permissive RXR-RAR heterodimer. RXR agonists alone thus cannot activate RXR-RAR heterodimer [11, 14], but can activate the heterodimer only in the presence of RAR agonists [34]. Therefore, the anti-tumor effects of HX630 and PA024 might be mediated by active RXR homodimer or permissive heterodimers (e.g., RXR-PPAR, RXR-LXR). Indeed, PPARγ or LXRα/β are expressed in AtT20 cells [40–43], and several studies have reported that PPARγ agonists induced G0/G1 cell-cycle arrest and apoptosis, and suppressed ACTH secretion in AtT20 cells [42, 43]. Additionally, it has also been reported that an RXR agonist, AGN194204, potentiated the anti-proliferative and pro-apoptotic responses of breast cancer cell lines to PPAR agonists through RXR-PPAR heterodimer [44]. In contrast, an LXR agonist could increase the number of cells immunostained with anti-ACTH antibody in the mouse pituitary [40]. Therefore, active RXR homodimer or RXR-PPAR heterodimer might be mainly associated with the anti-tumor effects of HX630 and PA024. Although the molecular mechanisms of their anti-tumor effect remain unclear, the microarray analyses showed that HX630 regulated the expression of a large number of molecules associated with cell death or growth, leading to tumor suppression. Among them, apoptotic pathways are speculated to play a crucial role in the anti-tumor effect of HX630, since the significant gene network leading to caspase 3 activity was identified from the microarray analyses. Further studies are required to elucidate the precise RXR signaling pathways and the subsequent molecular mechanisms of the anti-tumor effect by HX630.

On the other hand, we also demonstrated that HX630 decreased the *Pomc* promoter activity, *Pomc* mRNA expression, and ACTH secretion dose-dependently in AtT20 cells, while PA024 did not affect them. The HX630-mediated suppression of the *Pomc* promoter activity was demonstrated to be mediated via RXRα, although the *Pomc* 5’-flanking region (-703 to +58) used did not contain any RXR binding elements including direct repeat (DR) 1, DR 4, and DR5 [11, 12]. Therefore, HX630 may affect the expression of genes upstream of *Pomc* including transcription factors via RXR. The functional analyses of the *Pomc* promoter activity using *Pomc* 5’-flanking region deletion mutants indicated that several transcription factors affecting the promoter region might be involved in the HX630-mediated transcriptional suppression. Indeed, there are several binding sites of transcription factors in the *Pomc* promoter region [45]. Among them, there are two Nur DNA binding sites identified on the *Pomc* promoter. The proximal binding sequence termed Nur77-binding response element (NBRE) (-69/-63) binds to Nur77 monomer [45, 46]. On the other hand, the distal Nur response element (NurRE), constituted of two inverted NBRE related sites (-404/-397 and -390/-383), binds to Nur77 homodimer or Nur77/Nurr1 heterodimer, and NurRE responds to Nur77 much stronger than the proximal NBRE [29, 30, 45]. Furthermore, there are other transcription factor binding elements, E-box (-377/-370) [31, 32, 45], Tpit/Pitx-responsive element (-316/-309, -302/-297) [45, 47], and NF-κB-responsive element (-151/-142) [45, 48]. There has been no report showing the involvement of transcription factors in the *Pomc* promoter activity mediated by RXR agonists, although a natural RAR agonist, ATRA, inhibited ACTH secretion *in vitro* by inhibiting the transcriptional activity of the transcription factors Nur77/Nurr1 on the *Pomc* promoter [4]. In the present study, we demonstrated for the first time that HX630 negatively regulated the *Pomc* promoter activity at the transcriptional level due to the suppression of *Nur77* and *Nurr1* mRNA expression and the reduction of Nur77/Nurr1 heterodimer recruiting to NurRE in the *Pomc* promoter region. Nur77/Nurr1 heterodimer was reported to enhance NurRE transcription synergistically in comparison to that seen with Nur77 homodimer [30]. Therefore, these results indicate that Nur77/Nurr1 heterodimer is crucial for the molecular pathway involved in the HX630-mediated suppression of the *Pomc* promoter activity, resulting in the suppression of *Pomc* mRNA expression and ACTH secretion in AtT20 cells.

There were differences in the effects on *Pomc* promoter activity, *Pomc* mRNA expression, and ACTH secretion between HX630 and PA024, although they are both RXR-selective pan-agonists. RXRs interact with various factors, including heterodimer partners of nuclear receptors, co-repressors, and co-activators. Owing to such complex protein-protein interactions, different RXR agonists do not necessarily exhibit the same biological activities [20]. For example, it was reported that PA024 potently induced ABC transporter A1 (*ABCA1*) expression in macrophage cell lines, RAW264 cells and undifferentiated THP-1 cells through LXR-RXR heterodimer activation, while HX630 failed to induce *ABCA1* expression because of the inability to activate LXR-RXR heterodimer. Instead, HX630 was able to activate PPARγ/RXR, and induced *ABCA1* expression in differentiated THP-1 cells [49]. Another study demonstrated that combinations of Am80 with HX630 or with PA024 showed different gene expression profiles in microarray during the induction of HL-60 cell differentiation, and that the combination of Am80 with PA024, but not with HX630, especially increased the induction of HL-60 cell apoptosis, indicating that these two retinoid synergists might differently modulate the activation procedure of the RAR-RXR heterodimer [50]. The differential effects of HX630 and PA024 on RXR homodimer/RXR-RAR heterodimer transcription that we observed may also be involved in their differential effects on *Pomc* expression and ACTH secretion.

In conclusion, we demonstrated that HX630 at high concentrations suppressed cell proliferation and induced apoptosis, most likely through the activation of caspase 3, in AtT20 cells. We also provide new evidence that HX630 negatively regulates the *Pomc* promoter activity at the gene transcription level due to the suppression of *Nur77* and *Nurr1* mRNA expression and the subsequent reduction of Nur77/Nurr1 heterodimer recruiting to the *Pomc* promoter region, resulting in the suppression of *Pomc* mRNA expression and ACTH secretion. These effects of HX630 were shown to be exerted via RXRα. Furthermore, we confirmed that HX630 indeed inhibited tumor growth and decreased *Pomc* mRNA expression in corticotroph tumor cells in female nude mice *in vivo*. Thus, these results indicate that RXR agonists, especially HX630, might be a new therapeutic candidate for Cushing’s disease. Further structural optimization of them to reduce the effective concentration for decreasing *Pomc* expression and ACTH secretion, inducing apoptosis, and inhibiting cell proliferation may be necessary for the introduction into the clinical application.

## Supporting Information

S1 File NC3Rs ARRIVE Guidelines Checklist. (DOC)
